# Extracellular vesicles: a promising cell-free therapy for cartilage repair

**DOI:** 10.2144/fsoa-2021-0096

**Published:** 2021-12-06

**Authors:** Rizka Musdalifah Amsar, Christofora Hanny Wijaya, Ika Dewi Ana, Atik Choirul Hidajah, Hari Basuki Notobroto, Triati Dewi Kencana Wungu, Anggraini Barlian

**Affiliations:** 1 School of Life Science & Technology, Institut Teknologi Bandung, Bandung, West Java, 40132, Indonesia; 2 Department of Food Science & Technology, Bogor Agricultural University, West Java, 16680, Indonesia; 3 Department of Dental Biomedical Sciences, Faculty of Dentistry, Gadjah Mada University, Yogyakarta, 55281, Indonesia; 4 Department of Epidemiology Faculty of Public Health, Airlangga University, East Java, 60115, Indonesia; 5 Department of Biostatics & Population Faculty of Public Health, Airlangga University, East Java, 60115, Indonesia; 6 Nuclear Physics & Biophysics Research Group, Department of Physics, Faculty of Mathematics & Natural Sciences, Institut Teknologi Bandung, West Java, 40132, Indonesia; 7 Research Center for Nanoscience & Nanotechnology, Institut Teknologi Bandung, West Java, 40132, Indonesia

**Keywords:** cartilage repair, chondrocyte, exosomes, extracellular vesicles, stem cell, therapeutic strategies

## Abstract

Few effective therapies for cartilage repair have been found as cartilage has a low regenerative capacity. Extracellular vesicles (EVs), including exosomes, are produced by cells and contain bioactive components such as nucleic acids, proteins, lipids and other metabolites that have potential for treating cartilage injuries. Challenges like the difficulty in standardizing targeted therapy have prevented EVs from being used frequently as a treatment option. In this review we present current studies, mechanisms and delivery strategies of EVs. Additionally, we describe the challenges and future directions of EVs as therapeutic agents for cartilage repair.

## Lay abstract

Repairing cartilage damage is challenging due to the tissue’s low regenerative capacity. Extracellular vesicles (EVs) contain bioactive components that may be able to treat cartilage injuries. However, EV-based therapy is not widely used. This review summarizes the current state of knowledge regarding the use of EVs for cartilage repair, including the mechanisms, delivery strategies, challenges and future directions.

Cartilage is a type of connective tissue in the body that contains extracellular matrix and chondrocytes. Cartilage damage can be caused by both degenerative disease and trauma. Treatment of cartilage damage remains challenging due to the nature of the tissue, which does not readily regenerate. Cartilage is avascular, alymphatic and aneural. Osteochondral grafts, collected from bone and intact articular cartilage from a non-weight-bearing portion of the knee, can be used to treat cartilage damage in a weight-bearing site. Microfracture, another therapy, is based on cell homing [1]. Microfractures are created at 3- to 4-mm intervals, stimulating production of a blood clot containing bone marrow stem cells. The stem cells will differentiate into chondrocytes and secrete extracellular matrix (ECM) to produce cartilage. Cell-based therapy such as autologous chondrocyte implantation (ACI) is used to treat cartilage defects [2]. ACI includes chondrocyte isolation, *in vitro* culture and implantation into the injury site. ACI can be modified by using chondrocytes seeded in matrix (matrix-associated ACI) to improve cell delivery. The source availability, risk of graft rejection and formation of fibrocartilage rather than hyaline cartilage are all issues with current treatment methods [3].

Stem cells are commonly used as therapeutic cells in tissue regeneration. Stem cells can be implanted directly at injury sites or used as the cell source for tissue engineering [4]. Stem cells are more readily available than chondrocytes. Stem cells needed to treat damaged cartilage can be obtained from induced pluripotent stem cells [5], amniotic fluid stem cells (AFSCs) [6], Wharton jelly-derived stem cells [7], adipose-derived stem cells (ADSCs) [8] and bone marrow-derived stem cells (BMSCs) [9]. Stem cells present some advantages in cell-based therapies. For instance, mesenchymal stem cells (MSCs) can proliferate and differentiate into specific cell types and replace the targeted affected tissue [10]. Another advantage of using MSCs in therapy is that the cells participate in immunomodulation [11].

Recent studies have attributed the value of stem cells in therapy to their paracrine secretion [12]. Cells release paracrine factors via extracellular vesicles (EVs) such as exosomes. Exosomes function in cell–cell communication and can be found in all bodily fluids, including milk [13], urine [14], blood [15] and saliva [16]. Their cargo depends on their cell of origin [17]. Exosomes have potential uses in the repair and regeneration of damaged cartilage [18,19]. Exosomes stimulate cell proliferation [20] and stem cell differentiation [21]. They also modulate inflammation in injured cartilage [22]. The use of exosomes in cartilage repair can minimize immune rejection [23] . In addition, exosome treatment may result in the formation of hyaline cartilage. Zhang *et al*. demonstrated that intra-articular injection of exosomes weekly for 12 weeks completely restored cartilage and subchondral bone with hyaline cartilage in an osteochondral defect model [24].

EVs’ presence and therapeutic function allow for clinical applications in cartilage repair and regeneration. However, the delivery strategy for their use as a targeted therapeutic agent is challenging. EVs have a variable protein or nucleic acid profile, and in small amounts they can be cleared rapidly by the circulatory system.

Here we provide information about EVs, current studies on the potential use of EVs/exosomes for cartilage regeneration, and therapeutic strategies for using EVs as well as their limitations.

## EVs & exosomes

Most cells spontaneously secrete vesicles into the extracellular space. EVs are defined as lipid bilayer particles released by cells that are unable to multiply, according to the Minimal Information for Studies of Extracellular Vesicles 2018 (MISEV2018) [25]. Based on their biogenesis, size and content, EVs can be categorized into three types: apoptotic bodies, microvesicles/shedding particles and exosomes [26,27]. During the apoptotic process, dying cells form vesicles called apoptotic bodies. The origin of apoptotic bodies is the outward blebbing (1–5000 nm) of the apoptotic cell membrane [28], and the vesicles are formed to enhance removal of apoptotic material [27]. Microvesicles or exosomes are heterogeneous vesicles with a size range from 100 to 500 nm [29]. Microvesicles are derived from outward budding from the plasma membrane [30]. Exosomes, derived from endosomal origins, are the smallest type of EVs, with sizes ranging from 30 to 100 nm [31–33]. Due to the distinct biogenesis pathway, the molecular profile varies between each type of EV. For instance, the types of protein and lipid content in microvesicles and exosomes are different [34].

The formation of exosomes begins with inward budding of the cell membrane and the production of early endosomes. In the cytosol, early endosomes develop into late endosomes and make multivesicular bodies (MVBs) [35]. The invagination of the MVB membrane produces intraluminal vesicles. There are two usages of MVB in the cytosol: fusion to lysosomes for degradation, or fusion with the cell membrane to release intraluminal vesicles as exosomes [29]. Tetraspanins such as CD63, CD9 and CD81 are used as exosome markers in many studies; however, tetraspanin is also present on the cell surface, while other types of EVs have the marker in their membrane [36]. Because specific exosome markers have not yet been established, the MISEV2018 suggested operational terms for EV subtypes that refer to physical characteristics, biochemical composition and the cell origin of EVs [25]. This review uses the term EV to indicate general vesicles produced by cells, including exosomes.

The composition of lipids in the EV membrane resembles that of the cell plasma membrane [37]. However, Llorente *et al*. reported that exosomes have the highest content of glycosphingolipids, sphingomyelin, cholesterol and phosphatidylserine compared with the parent cell [38]. Lipids in the EV membrane maintain the EV’s stability in the extracellular environment and facilitate uptake into recipient cells [37,39]. Protein from the EV membrane plays a role in tissue repair [40]. Moreover, proteins in the EV membrane contribute to the interaction between EVs and recipient cells. EV cargo also includes nucleic acids such as DNA and miRNA, which is the most studied nucleic acid. In cartilage repair and regeneration, exosomal RNA regulates genes involved in inflammation, cell proliferation, apoptosis and ECM synthesis. Mao *et al*. demonstrated that during 14 days of chondrogenesis, MSCs treated with 100 µg/ml exosomal *circ_0001236* expressed more *SOX9* and *COL2A1* than MSCs treated with 50 µg/ml exosomal *circ_0001236* [41]. *SOX9* and *COL2A1* are markers of chondrogenic differentiation, and this study demonstrated that exosomal *circ_0001236* at higher concentrations enhanced chondrogenesis in MSCs.

EVs may interact with recipient cells via contact, membrane fusion or endocytosis as a mediator of intercellular communication. In contact, the membrane ligand on the EVs’ surface interacts with the receptor in the cell membrane of the recipient cell and generates cell signals [42,43]. In this case, EVs will not be internalized by the target cells. Another mechanism of interaction between EVs and cell targets is membrane fusion, in which the EV membrane consists of a lipid bilayer that fuses with the cell membrane and releases the cargo into the cytosol. A study by Parolini *et al*. showed that exosome uptake by melanoma cells happened via fusion and increased at low pH [44]. The most commonly studied mechanism of EV internalization is the endocytosis pathway, in which the EVs enter the recipient cell by phagocytosis [45], macropinocytosis [46,47], clathrin-mediated endocytosis [46], caveolin-mediated endocytosis [48] or lipid raft-mediated endocytosis [49]. It is possible that a particular type of EV has more than one mechanism when interacting with a recipient cell. Because EVs have therapeutic potential, their interactions with cells should be studied to develop targeted therapies.

Stem cell-derived EVs are at least as good as, if not better than, stem cells when applied for therapeutic purposes. Overall, they demonstrate less negative potential. A study by Mohammed *et al*. showed that exosomes from ADSCs are more effective as an adjuvant treatment in dentistry for scaling and root planing [50]. Another study performed by Zavatti *et al*. compared AFSCs and their exosomes in animal models of osteoarthritis and found that AFSC-derived exosomes were more effective in treating cartilage damage than the cells [51]. When compared with cell-based therapy using stem cells, EVs have some distinct advantages. For example, EVs have simpler storage needs, allow allogeneic transplantation due to lack of MHC I and MHC II antigens and are less vulnerable to damage at the injury site; it is also possible they can reach a higher circulating dose than bigger cells [23]. Because EVs are non-self-replicating, the possibility of iatrogenic tumor growth is reduced.

MSC exosomes are effective in supporting cartilage repair and regeneration [52]. The application of EVs in cartilage repair has been investigated *in vivo* in many different animal models with a variety of concentrations (Table 1). Small animal models such as mice, rats and rabbits are used in current research on cartilage regeneration. However, more research with larger test animals is required to be clinically appropriate. To improve treatment efficacy, it will also be necessary to standardize the EVs dose calculation.

**Table 1.** A summary of *in vivo* studies that use extracellular vesicles for cartilage repair.

Animal model	Source of extracellular vesicle	Dose	Delivery	Ref.
OA induce in mice	BMSC	500 μg/ml	Intra-articular injection	[41]
OA induced in rat	BMSC	400 μg/ml	Intra-articular injection	[53]
OA induced in rat	SMSC	10^11^ particles/ml	Intra-articular injection – scaffold PLEL	[54]
Mice defect model	L-cells	7 μL	Intra-articular injection	[55]
Rabbit defect model	IPF-MSC	10^10^ particles	Intra-articular injection	[56]
Rat	CESC	10^5^ particles/ml	Intradiscal injection	[57]
Rabbit osteochondral defect model	WJ-MSC	25 μg/ml	Injection	[58]
Rat defect model	UMSC	1 mg/ml	Injection	[59]
OA induced in rat	BMSC	10^10^ particles/ml	Intra-articular injection	[60]
Rat defect model	UMSC	1 mg/ml	Intra-articular injection	[61]
OA induced in rat	BMSC	1 µg/μl	Injection	[62]
OA induced in rat	Dendritic cell (kartogenin)	100 μl	Intra-articular injection	[63]
Rat defect model	UMSC	10^8^ particles/ml	With scaffold implant directly	[64]
OA induced in rat	BMSC	40 μg/100 μl	Intra-articular injection	[65]
Rabbit osteochondral defect model	Embryonic stem cell-derived MSC	200 μg/mL of 3% HA	Intra-articular injection	[66]
OA induced in mice	Chondrogenic progenitor cell	10^10^ particles/ml	Intra-articular injection	[67]
Rabbit defect model	UMSC	10^10^ particles/ml	Intra-articular injection	[68]
OA induced in rat	AFSC	2 μg/μl	Unilateral injection	[51]
OA induced in mice	BMSC	1 μg/μl	Tail vein injection	[69]
IVD degeneration rabbit model	BMSC	1 μg/μl	Intradiscal injection	[70]
Rabbit defect model	BMSC	200 μg/ml	Implantation of ECM/GelMA/exosome scaffold	[71]
OA induced in mice	IPF-MSC	10^10^ particles/ml	Intra-articular injection	[72]
OA induced in rat	MSC	10^11^ particles/ml	Articular cavity injection	[73]
OA induced in rat	Embryonic stem cell-derived MSC	2 μg/μl	Intra-articular injection	[74]
OA induced in rat	SMSC	10^11^ particles/ml	Articular cavity injection	[75]
Rat osteochondral defect model	Embryonic stem cell-derived MSC	1 μg/μl	Intra-articular injection	[24]

AFSC: Amniotic fluid stem cell; BMSC: Bone marrow mesenchymal stem cell; CESC: Cartilage endplate stem cell; ECM: Extracellular matrix; GelMA: Gelatin methacrylate; HA: Hyaluronic acid; IPF-MSC: Infrapatellar fat pad mesenchymal stem cell; IVD: intervertebral disc; OA: Osteoarthritis; MSC: Mesenchymal stem cell; PLEL: poly(D,L-lactide)-b-poly(ethylene glycol)-b-poly(D,L- lactide; SMSC: Synovial mesenchymal stem cell; UMSC: Umbilical cord mesenchymal stem cell; WJ-MSC: Wharton Jelly mesenchymal stem cell.

## Sources of EVs

EVs can be obtained from almost all bodily fluids. Parental cell selection should account for the desired therapeutic function of the resultant EVs. EVs can be a therapeutic drug or they can act as a delivery vehicle for a specific drug. For instance, EVs isolated from bovine milk can be utilized to deliver exogenous hsa-miR148a-3p in RNA-based treatment [76]. Although cells from an injury site can produce EVs, they usually do not produce therapeutic EVs; rather, EVs from the cells in a cartilage injury site tend to aggravate the damage [77,78]. However, EVs from therapeutic cells at the same injury site can maintain chondrocyte homeostasis [78]. These therapeutic cells can be differentiated cells or stem cells. Ma *et al*. found that EVs released by chondrocytes induced proliferation and differentiation of umbilical cord MSCs into chondrocytes, indicating that EVs promote cartilage regeneration [79].

EVs from blood components are advantageous because blood collection is less invasive and safer than adipose tissue or bone marrow collection. Otahal *et al*. studied the use of EVs derived from blood for treatment of osteoarthritis [80]. These investigators demonstrated that EVs isolated from citrate-anticoagulated platelet-rich plasma-enhanced desirable chondrogenic gene expression changes in osteoarthritis and prevented proinflammatory cytokine release [80]. Another study by Liu *et al*. showed that EVs derived from platelet-rich plasma promoted proliferation and inhibited chondrocyte apoptosis via the Wnt/β-catenin signaling pathway [81].

Stem cells such as induced pluripotent stem cells and MSC have potential in tissue repair. As cell-based therapy, stem cells can be applied directly or serve as a cell source for tissue engineering. Various types of stem cells produce functional EVs with advantages for cartilage repair. EVs derived from AFSCs can repair cartilage damage in correlation with their TGF-β content [51]. MSCs, which are non-hematopoietic stem cells, are present in various body tissues and are multipotent. The therapeutic effect of MSCs depends on a paracrine mechanism mediated by their EVs [12]. EVs isolated from ADSCs prevent cartilage degeneration and attenuate the progression of osteoarthritis by modulating immune reactivity [20]. Another study, using EVs from BMSCs, showed that BMSC-derived EVs promote ECM synthesis and protect against cartilage damage [65]. MSC-derived exosomes promote proliferation, migration and ECM synthesis, which helps to attenuate apoptosis and modulates immune reactivity in osteochondral defects [82].

Further research is needed to determine the most efficient therapeutic cell source, propagation and storage methods. An *ex vivo* study performed by Li *et al*. compared EVs from ADSCs, BMSCs and synovium MSCs in cartilage regeneration and demonstrated that ADSC-derived EVs are the best candidate for cartilage and bone regeneration [83]. Even though that study was conducted *ex vivo*, it reveals that EVs derived from different cell types have variable effects. Moreover, a proteomic analysis of exosomes isolated from BMSCs, ADSCs and umbilical cord MSCs demonstrated their potential utility in a variety of fields [84].

## Mechanism of EVs in cartilage regeneration

Common causes of cartilage damage are trauma and degenerative disease. In articular cartilage, damage often results from violent injury, chronic inflammatory disease or degenerative joint diseases [85]. According to Schulze-Tanzil [86], traumatic cartilage injury causes chondrocyte and synoviocyte stress that leads to inflammation, degradation of the cartilage’s ECM and apoptosis. Inflammation in cartilage is often caused by inflammatory cytokines including IL-1β, TNF-α, IL-6, IL-15, IL-17 and IL-18 [87]. Cartilage damage has an effect on the quantity of chondrocytes by triggering cell death [88] and inducing chondrocyte apoptosis [89]. Additionally, injured ECM degrades faster than it can be synthesized. Understanding the pathogenesis of cartilage injury can help scientists develop specific therapies, including therapy for cartilage damage directed to overcome the results of homeostatic changes.

Inflammation in cartilage tends to increase pain and disease progression. Inflammation is a phenomenon in traumatic cartilage injury [86]. If the damage is caused by degenerative disease, such as in the intervertebral disc (IVD), inflammation is caused by an imbalance of the ECM catabolic and anabolic pathways [90]. Treatment using EVs can inhibit the inflammatory cascade. A study by Zhang *et al*. indicated that MSC-derived exosomes reduced IL-1β [74]. IL-1β, as the most important proinflammatory mediator, is also involved in inflammatory responses during disc degeneration [91]. Another study showed that MSC-derived exosomes slowed the progression of IVD degeneration by suppressing inflammatory mediators and NLRP3 inflammasome activation [70]. Suppressing the NLRP3 pathway can prevent pyroptosis, a programmed cell death triggered by proinflammatory signals. Exosomal miR-410 from MSC inhibits the NLRP3 pathway and regulates pyroptosis [92]. Recently, treatments for osteoarthritis have focused on macrophage polarization. Macrophages are immune cells found in the synovial lining that complete a variety of tasks depending on their subtype; they may be proinflammatory (M1) or anti-inflammatory (M2) [93]. A study by Zhang *et al*. demonstrated that exosomes isolated from BMSCs reduced inflammation by regulating macrophage polarization, inhibiting M1 macrophage production and promoting M2 macrophage generation [60].

The purpose of therapy in cartilage repair is to restore the chondrocyte ECM to its original state. ECM components, like collagen type II and proteoglycan, play a role in regulating chondrocyte functions. Therapy can be aimed at synthesizing those specific ECMs. He *et al*. reported that BMSC-derived exosomes upregulated collagen type II production and downregulated MMP13 protein expression in an animal model of osteoarthritis [65]. Another study found that BMSC-derived exosomes promoted ECM production in degenerated nucleus pulposus cells *in vitro* [94]. Thus it appears that EVs play defined roles in recovering cartilage ECM.

Chondrocytes play a role in cartilage regeneration by synthesizing ECM, despite their low number in normal cartilage. Because cartilage injury further diminishes the number of chondrocytes, a therapeutic method is required to maintain their population. The number of chondrocytes can be maintained by several mechanisms, one of which is to differentiate stem cells into chondrocytes. EVs from nucleus pulposus cells induce differentiation of MSC into nucleus pulposus-like cells by inhibiting the Notch1 pathway [95]. One component of EVs, miRNA, can also target the pathway in chondrogenic differentiation. Li *et al*. showed that miR-8485 from exosomal chondrocytes activated the Wnt/β-catenin pathways to stimulate differentiation of BMSCs into chondrocytes [96]. In addition to chondrogenic differentiation of stem cells, increasing chondrocyte proliferation in the injury site improves cartilage regeneration. Some studies have shown that EV cargo – for instance, miRNA [61,62,97 – can promote chondrocyte proliferation.

Chondrocyte loss that is caused by apoptosis and autophagy can be overcome using EV therapy. Cheng *et al*. reported that miR-21 in MSC-derived exosomes prevented nucleus pulposus cell apoptosis [98]. Similarly, studies have shown the utility of EVs in the inhibition of apoptosis induced by endoplasmic reticulum in IVD degeneration [99,100]. Inhibiting apoptosis and increasing cell proliferation in cartilage repair will maintain the number of chondrocytes.

**Figure 1.**
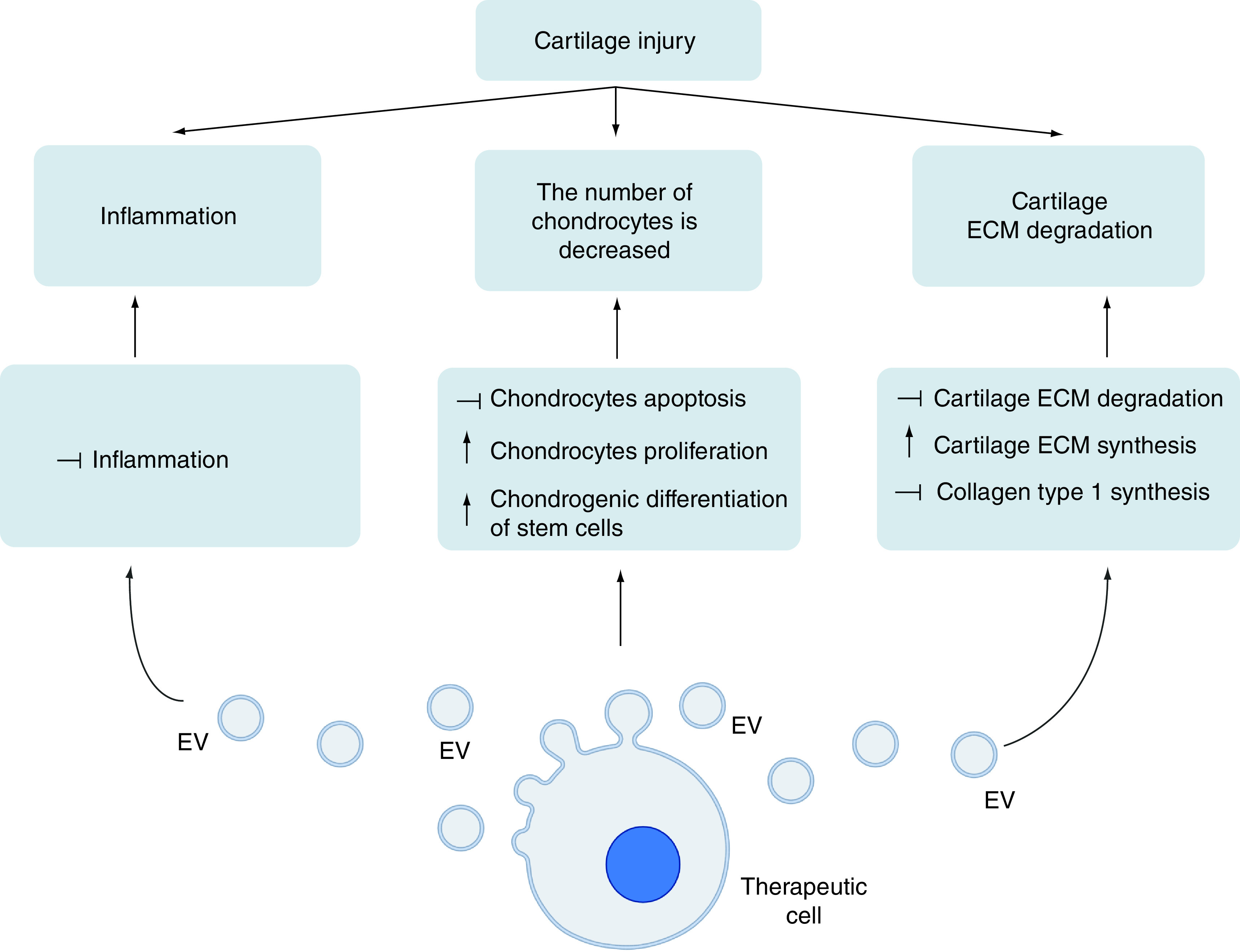
Mechanism of EVs in cartilage repair. Inflammation, chondrocyte reduction, and cartilage extracellular matrix degradation are the three phenomena that occur in cartilage damage. Extracellular vesicles work to overcome this by releasing cargo that can lower inflammation, increase the number of chondrocytes, and restore cartilage extracellular matrix in injury sites. (Created with BioRender.com). ECM: Extracellular matrix; EV: Extracellular vesicle.

The role of EVs in cartilage treatment is to restore cartilage homeostasis by maintaining the number of chondrocytes and balancing the metabolism of specific ECMs (Figure 1). EVs deliver functional cargo, such as miRNA, for cartilage regeneration (Table 2). Additionally, EV cargo modulates inflammation at the injury site.

**Table 2.** Role and mechanism of exosome-derived RNA in cartilage regeneration.

Exosomes component	Donor cell	Target cell	Induced mechanism	Ref.
miR-216-5p	BMSC	Chondrocyte	Enhance chondrocyte proliferation, migration and apoptosis repression	[62]
lncRNA H19	UMSC	Chondrocyte	Promote proliferation and prevent apoptosis in chondrocytes	[61]
miR-8485	Chondrocyte	BMSC	Promote chondrogenic differentiation of BMSCs	[96]
mir-145 and mir-221	ADSC	Chondrocyte	Promote proliferation	[97]
miR-100-5p	IPF-MSC	Chondrocyte	Inhibit mTOR autophagy pathway	[72]
miR-92a-3p	BMSC	Chondrocyte, MSC	Promote chondrocyte proliferation and matrix genes expression	[101]

ADSC: Adipose-derived stem cell; BMSC: Bone marrow mesenchymal stem cell; IPF-MSC: Infrapatellar fat pad mesenchymal stem cell; MSC: Mesenchymal stem cell; UMSC: Umbilical cord mesenchymal stem cell.

## Delivery strategies of EVs in cartilage repair

It is necessary to design suitable EVs that are functional therapeutic agents and deliver them to enhance their effectiveness and efficiency in treating damage. For cartilage repair, EVs can be obtained from bodily fluid, tissue or cell culture and delivered by local or intravenous administration. Woo *et al*. isolated EVs from ADSCs and used them to treat osteoarthritis in rats [20]. They found that ADSC-derived EVs enhanced proliferation and migration of chondrocytes, regulated the expression of catabolic and anticatabolic factors and inhibited macrophage infiltration into synovium, thereby modulating immune reactivity [20]. He *et al*. also demonstrated that EVs derived from BMSCs and injected intra-articularly stimulated cartilage regeneration and ECM synthesis, as well as reducing knee discomfort, in an osteoarthritis model [65].

### Engineering cells & their EVs

Engineering parental cells or their EVs can enhance the effectiveness of EVs in therapy. Changes in the cell microenvironment – such as the pretreatment medium, oxygen level and mechanical stimulation – influence cell behavior and affect EV characteristics and functions. For instance, kartogenin has been used to improve stem cell proliferation and chondrogenic differentiation in cartilage regeneration [102]. An examination of EVs from cells pretreated with kartogenin revealed a paracrine change of the cells in chondrogenesis. Liu *et al*. reported that EVs derived from kartogenin-preconditioned BMSCs enhanced chondral matrix synthesis and reduced degradation; thus this approach appears more effective for cartilage repair than the use of EVs from BMSCs without pretreatment with kartogenin [103]. A study using infrapatellar fat pad MSCs showed a similar result: EVs pretreated with kartogenin more effectively promoted articular cartilage defect repair [56]. Thus by altering the cellular environment through the addition of chemical compounds to the cell culture medium, the efficiency of the resultant EVs is improved.

Hypoxic preconditioning of stem cells also affects the efficiency of EVs. Hypoxic pretreatment of BMSCs enhances their release of EVs that increase proliferation, migration and apoptosis inhibition of chondrocytes through the miR-216a-5p/JAK2/STAT3 signaling pathway [62]. The cell microenvironment can also be modified through mechanical stimulation. A study by Yan *et al*. showed that mechanical stimulation using a rotary cell culture system enhanced the yield of EVs from umbilical cord MSC-derived EVs and found that EV function on cartilage repair was enhanced through upregulation of lncRNA *H19* [61]. Modification of parental cells can also be accomplished by genetic engineering; Thomas *et al*. successfully engineered L-cells with WNT3a and isolated EVs that were able to heal osteochondral defects [55].

Another target of modification for targeted therapy, besides parental cells, is the EV itself, via a method called post-secretion modification. The aim of engineering EVs is to make them a functional drug delivery system. The drug loaded in EVs can be a natural component of therapeutic cell-derived EVs or another chemical agent. Loading a drug into EVs increases its *in vivo* stability, circulation in blood, and cell targeting efficiency [104]. Combining EVs with drugs promoting cartilage regeneration, such as kartogenin, enhances their function. Post-secretion modification of EVs is more efficient than engineering parental cells to deliver drugs. For example, even though kartogenin is beneficial in cartilage regeneration, it has low water solubility. Xu *et al*. isolated EVs from dendritic cells and engineered them to be a delivery agent for kartogenin [63]. They showed that this treatment increased the effectiveness of synovial fluid-derived MSCs to differentiate into chondrocytes [63]. Post-secretion modification can also be performed on the EV surface by adding specific ligands. Engineering the natural surface increased targeting efficiency *in vivo* [105].

For the same dose, delivering EVs through intravenous administration is less effective than local administration in cartilage repair. The half-life of exosomes in blood circulation is about 2 min [106]; healing a cartilage injury requires more time due to the characteristics of cartilage. However, local administration methods such as intra-articular injection require frequent injections that make the patient uncomfortable. Combining EVs with biomaterials or scaffolds could reduce treatment frequency, as the biomaterial will ensure that the EVs remain at the defect site.

### EVs embedded in biomaterials

The scaffold acts as a time-controlled delivery system for EVs in cartilage injury, trapping them at the injury site and periodically releasing them. The release of drugs or EVs from a scaffold can be caused by diffusion, polymer dissolution and degradation, or swelling [107]. Scaffolds are defined by their ability to retain EVs at the injury site, gradually release them into the matrix and integrate with the damaged tissue to promote surrounding cell migration [108].

Scaffolds for cartilage regeneration can be made from synthetic or natural materials. Some common synthetic polymer materials are poly(lactic-co-glycolic acid) and polymer of lactic acid [109]. Synthetic materials have the advantages of reproducibility, structure and customizable characteristics. However, synthetic materials are more expensive than natural ones and they have weak cell attachment [109]. Additionally, natural scaffolds such as collagen, fibroin and chitosan tend to be safer because of their biocompatibility and reduced toxicity. The drawbacks of natural scaffolds are their source-dependent mechanical and physical properties [110].

The scaffold form needed to trap EVs and maintain their release can be solid or hydrogel. Hydrogel, a hydrophilic polymer, is widely used in cartilage tissue engineering. Its mechanical behavior permits its use as an articular cartilage substitute [111]. Hydrogel can be fabricated from natural materials or synthetic polymers to mimic the natural ECM and will control the release of EVs embedded in it. Stem cell-derived EVs can be incorporated into a photo-induced crosslinking hydrogel to retain the exosomes inside and enhance cartilage repair [112]. Chen *et al*. showed that EV-impregnated scaffolds from the cartilage ECM and gelatin methacrylate hydrogel promoted cartilage regeneration [71]. Thus studies indicate that biomaterial has a significant role in the delivery of EVs for repairing cartilage damage. Further research should be conducted to explore the various biomaterials that may be used for EV delivery in cartilage repair and regeneration.

**Figure 2.**
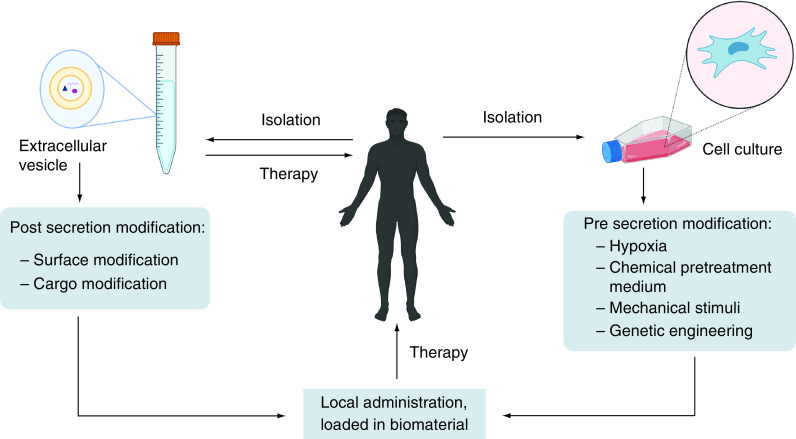
Strategies of extracellular vesicle based therapy in cartilage repair. EVs can be obtained from bodily tissue, fluid, or cell culture. Modification can be performed in cells (pre secretion of EVs) or EVs (post secretion). (Created with BioRender.com). EV: Extracellular vesicle.

Figure 2 summarizes therapeutic strategies utilizing EVs for cartilage repair. There are numerous delivery methods for EVs used to repair cartilage damage, with the primary goal being the restoration of cartilage homeostasis. The simplest method is to use naive EVs isolated from bodily tissue or cell cultures. However, EV parental cell types must be considered, as they affect EV bioactivity. By retaining EVs within the biomaterial and controlling their release, implanting EV-loaded biomaterials may be a way to enhance therapeutic effects. Furthermore, it does not require frequent administration when dosed appropriately.

## Limitations

EVs, particularly exosomes, have potential in cell-free therapy for cartilage repair and regeneration. Numerous *in vitro* and *in vivo* studies have delineated the composition of EVs and their role in tissue repair. However, a search of clinicaltrials.gov gave only one result, which involved the use of platelet-rich plasma enriched with exosomes in the treatment of chronic low back pain [113]. It is critical to have appropriate identity and potency parameters when studying EVs to ensure their quality control and reproducibility. Those studying EVs and their effects should refer to the International Society of Extracellular Vesicles’ guidelines [25] to promote reproducibility.

While evidence for the use of EVs in cartilage repair is convincing, several factors must be considered prior to initiating clinical trials. Larger animal models should be studied before EVs are used in the clinic. The examination of partial-thickness and full-thickness chondral repair, as well as osteochondral repair, is possible in large animal models with thicker articular cartilage [114]. Further investigation into the choice of EV parental cells and their maintenance is required due to the heterogeneity of EV content. As no single drug fits all diseases, targeted therapy is important. Another consideration is the optimization of large-scale production of EVs, because it is challenging to isolate EVs with high purity in high yields. The most common method for isolating EVs is ultracentrifugation, but this has limitations of low purity and EV aggregation [115]. Tangential flow filtration can be an alternative to achieve reproducible large-scale production [116]. Scale-up methods to produce EVs as a therapeutic agent for cartilage repair need standardization.

The choice of whether or not to engineer EVs for targeted cartilage therapy will depend in part on further research to guarantee their efficacy and safety. Proper EV dose and delivery strategies are also important. EVs wash out easily in the circulatory system, necessitating a higher dose or entrapment in biomaterial. Scaffold in the form of hydrogel is a good candidate as a delivery agent. While live cell transplantation is already widely used, EV-based therapy has a greater potential for repair due to the absence of cells. EV-loaded scaffolds can be adapted to the current surgical techniques applied to repair cartilage defects by implanting the EV-loaded scaffolds in the defect site. It is hoped that this procedure will eliminate the need for repeated operations by optimizing the EV dosage in the scaffold and will increase patient comfort. Additionally, when EVs are used therapeutically, such as in an articular cartilage injury, they can regenerate hyaline cartilage.

EV pharmacokinetics also needs to be considered for therapeutic development. Furthermore, it is necessary to standardize the quality of EVs as a product, such as storage conditions (e.g., temperature and expiration date). Although there are many challenges in the clinical application of EVs for cartilage repair, the evidence on the function of EVs in healing cartilage injury is promising. A better understanding of the potential of EVs in therapy and their greater accessibility may significantly reduce related healthcare costs.

## Conclusion & future perspective

EVs, including exosomes, can be obtained from any cell source. Determination of parental cells and therapeutic strategies are important in making EV therapy effective and efficient. EV-based therapy has the potential to repair cartilage damage by maintaining cartilage homeostasis. To optimize the therapeutic effects of EVs, they can be engineered and loaded with biomaterials to control their release. Proper strategies will lead to an increased accessibility and effectiveness of EV therapy for cartilage repair. To be clinically applicable, the standardization of EV products must be considered to ensure their safety.

**Executive summary**

- Extracellular vesicles (EVs), including exosomes, have the potential to treat cartilage damage by restoring cartilage homeostasis.
- Due to the heterogeneity of EV content, selection of parental cells and appropriate therapeutic strategies are important in targeted therapy.
- Loading biomaterials into EVs optimizes their effectiveness in cartilage repair.
- Some challenges in large scale production of EVs need to be addressed to facilitate their clinical application.
